# Ultra-wide-field fundus photography compared to ophthalmoscopy in diagnosing and classifying major retinal diseases

**DOI:** 10.1038/s41598-022-23170-4

**Published:** 2022-11-11

**Authors:** E. Midena, G. Marchione, S. Di Giorgio, G. Rotondi, E. Longhin, L. Frizziero, E. Pilotto, R. Parrozzani, G. Midena

**Affiliations:** 1 Department of Ophthalmology, University of Padova, Padova, Italy; 2 IRCCS–Fondazione Bietti, Rome, Italy

**Keywords:** Health services, Medical imaging, Retinal diseases, Biomarkers, Eye diseases

## Abstract

To analyze the performance of ultra-wide-field (UWF) fundus photography compared with ophthalmoscopy in identifying and classifying retinal diseases. Patients examined for presumed major retinal disorders were consecutively enrolled. Each patient underwent indirect ophthalmoscopic evaluation, with scleral depression and/or fundus biomicroscopy, when clinically indicated, and mydriatic UWF fundus imaging by means of CLARUS 500™ fundus camera. Each eye was classified by a clinical grader and two image graders in the following groups: normal retina, diabetic retinopathy, vascular abnormalities, macular degenerations and dystrophies, retinal and choroidal tumors, peripheral degenerative lesions and retinal detachment and myopic alterations. 7024 eyes of new patients were included. The inter-grader agreement for images classification was perfect (kappa = 0.998, 95% Confidence Interval (95%CI) = 0.997–0.999), as the two methods concordance for retinal diseases diagnosis (kappa = 0.997, 95%CI = 0.996–0.999) without statistically significant difference. UWF fundus imaging might be an alternative to ophthalmoscopy, since it allows to accurately classify major retinal diseases, widening the range of disorders possibly diagnosed with teleophthalmology. Although the clinician should be aware of the possibility that a minority of the most peripheral lesions may be not entirely visualized, it might be considered a first line diagnostic modality, in the context of a full ophthalmological examination.

## Introduction

The recent coronavirus disease 2019 (COVID-19) pandemic has forced the medical community to revise and reorganize the methods of clinical evaluation, particularly in ophthalmology: despite being the current gold standard for the examination of retinal periphery, indirect ophthalmoscopy, as well as central fundus biomicroscopy for macular disorders, require a close contact with the patient and need adequate time of evaluation^1–3^. The broad use of ultra-wide-field (UWF) fundus cameras has helped to partly overcome these obstacles, allowing: the clinician to analyze fundus images even at distance, namely in a different location than the clinic, to reduce the time of evaluation for the patient and the clinician; to perform screening for retinal disorders, such as diabetic retinopathy^4–6^ and peripheral retinal lesions^7–9^. Fundus imaging can be considered UWF when covering a field of retina equal to 100° or more^4,7,10–12^, showing, in a single shot, retinal features anterior to vortex vein ampullae in all four quadrants^13^. Conversely, the term “wide field” should be used for images showing retinal features beyond the posterior pole but posterior to vortex vein ampulla, in all four quadrants^13^.

The most widespread UWF fundus imaging systems currently in use are: Clarus™ (CLARUS 500™, Carl Zeiss Meditec AG, Jena, Germany) and Optos® (Optos California®, Optos PLC, Dunfermline, United Kingdom). Clarus™ is a fundus camera providing a real color photograph of retina and covering up to 133° of field in a single image, reaching over 200° of field with the auto-montage function. Eyelashes and eyelids artifacts are reduced or abolished thanks to the partially confocal optics of Clarus™. Some tools intrinsic to the system allow the clinician to compare images^4^. Conversely, Optos® is a scanning laser ophthalmoscope able to capture in a single image up to 200° of the retina. The combination of monochromatic red and green scanning laser leads to a semi-realistic two-tone fundus image which may show some differences with a real color picture^4^. These two cameras have shown similar effectiveness in grading the severity of diabetic retinopathy^4,14^, while it is still debated their role in localizing peripheral lesions^7–9^. Another fundus camera recently developed is Eidon (Centervue S.P.A., Padova, Italy), which is a confocal scanning laser ophthalmoscope, arranging a real fundus color image, encompassing a field of 90° in single exposure and up to 160° with mosaic function^15^.

The aim of this study was to assess the performance of one UWF fundus camera, namely Clarus™, compared to indirect ophthalmoscopy, plus central fundus biomicroscopy when macular involvement is suspected or present, in identifying and classifying major retinal disorders.

## Methods

### Population and procedures

This was a non-interventional cross-sectional study with prospective enrollment, compliant with the tenets of the Declaration of Helsinki and approved by the local Institutional Review Board (“Comitato Etico per la Sperimentazione Clinica della Provincia di Padova”—Prot. N. 11,870-2022). Informed consent was obtained from each patient. All new patients addressed to our Department and evaluated between February 2020 and December 2021 were consecutively included and examined for major retinal diseases. Each patient underwent indirect ophthalmoscopic evaluation, with scleral depression, when clinically indicated according to the examiner’s judgment, and/or central fundus biomicroscopy, when macular involvement was suspected or present, performed by a retinal (blinded to patient’s clinical records) expert and mydriatic UWF fundus imaging by means of Clarus™ fundus camera, perfomed by a specialized technician. Images were obtained for each gaze position (superior, inferior, right, left), auto-mounted by the system and exported for analysis as JPG files of 6604 × 4274 pixels. Pictures were then independently reviewed and classified by two masked retinal specialists, in order to calculate the intergrader agreement. The examiners were masked to each other evaluations and patients’ characteristics, including visual symptoms.

**Figure 1:**
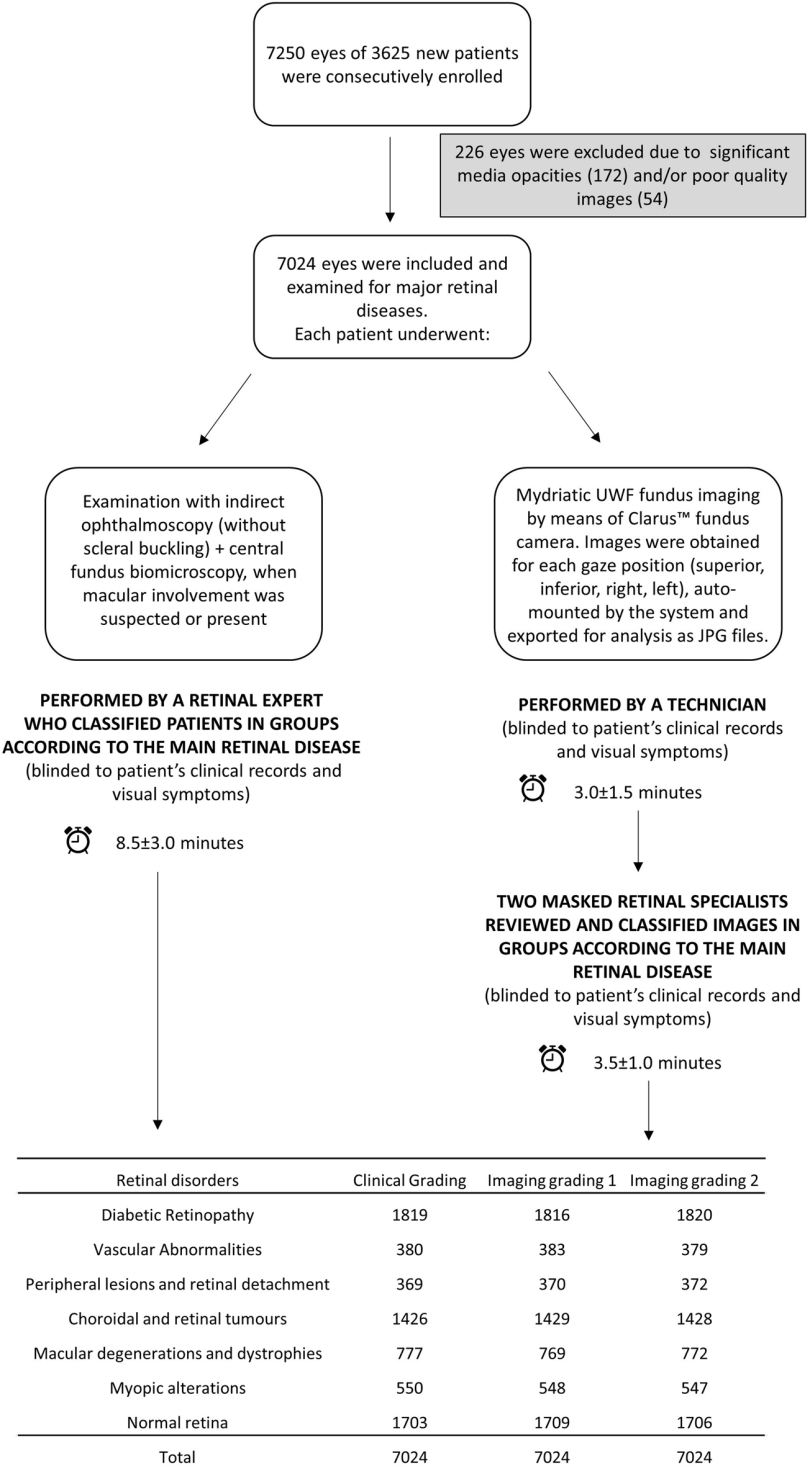
Flow chart detailing patients enrollment, images acquisition and classsification in the present study.

Thus, each eye received three independent classifications by: one clinical grader (CG) and two clinical image graders (IG1, IG2)^5,16^, in the following groups: normal retina (NR), diabetic retinopathy (DR), vascular abnormalities (VA), macular degenerations and dystrophies (MD), retinal and choroidal tumors (T), peripheral degenerative lesions and retinal detachment (PLD) and myopic alterations (MY). Graders were specifically trained to choose only one diagnosis, applying the most appropriate to each case. Poor quality images, patients with significant media opacities (corneal opacities, cataract or vitreous hemorrhage) were excluded. See Fig. 1 for the flow chart of the study.

### Statistical analysis

The sensitivity for identifying major retinal diseases with Clarus™ imaging system was calculated for each individual disorder as follows: number of eyes accurately classified on UWF imaging divided by number of eyes correctly labeled by ophthalmoscopy^8^. Specificity was calculated for each disease group as the number of not affected eyes identified by UWF imaging divided by those diagnosed as not affected by ophthalmoscopy. We considered statistical tests significant for p values less than 0.05. Both intergrader agreement and the consensus between clinical and imaging classification were quantified by the proportion of observed agreement (number of eyes for which the two assessments coincide on the total number of eyes evaluated), the simple and weighted kappa (k) and its 95% confidence interval (95%CI). Bias and prevalence index were also calculated, k coefficient by means of Prevalence-adjusted and bias-adjusted kappa coefficient (PABAK) and Gwet’s first-order agreement coefficient as well. Indexes of agreement were assessed both on overall results and for individual disorder. The interpretation of k value and of the other indexes was made according to the indication of Landis & Koch^17^: poor if k < 0, slight if 0–0.20, fair if 0.21–0.40, moderate if 0.41–0.60, substantial if 0.61–0.80, almost perfect if 0.81–1.00. All analyses were performed using SAS® v. 9.4 (SAS Institute, Cary NC, USA) on a personal computer. The SAS code macro provided by Yang and Zhou^18^ was used for the calculation of k.

## Results

**Figure 2:**
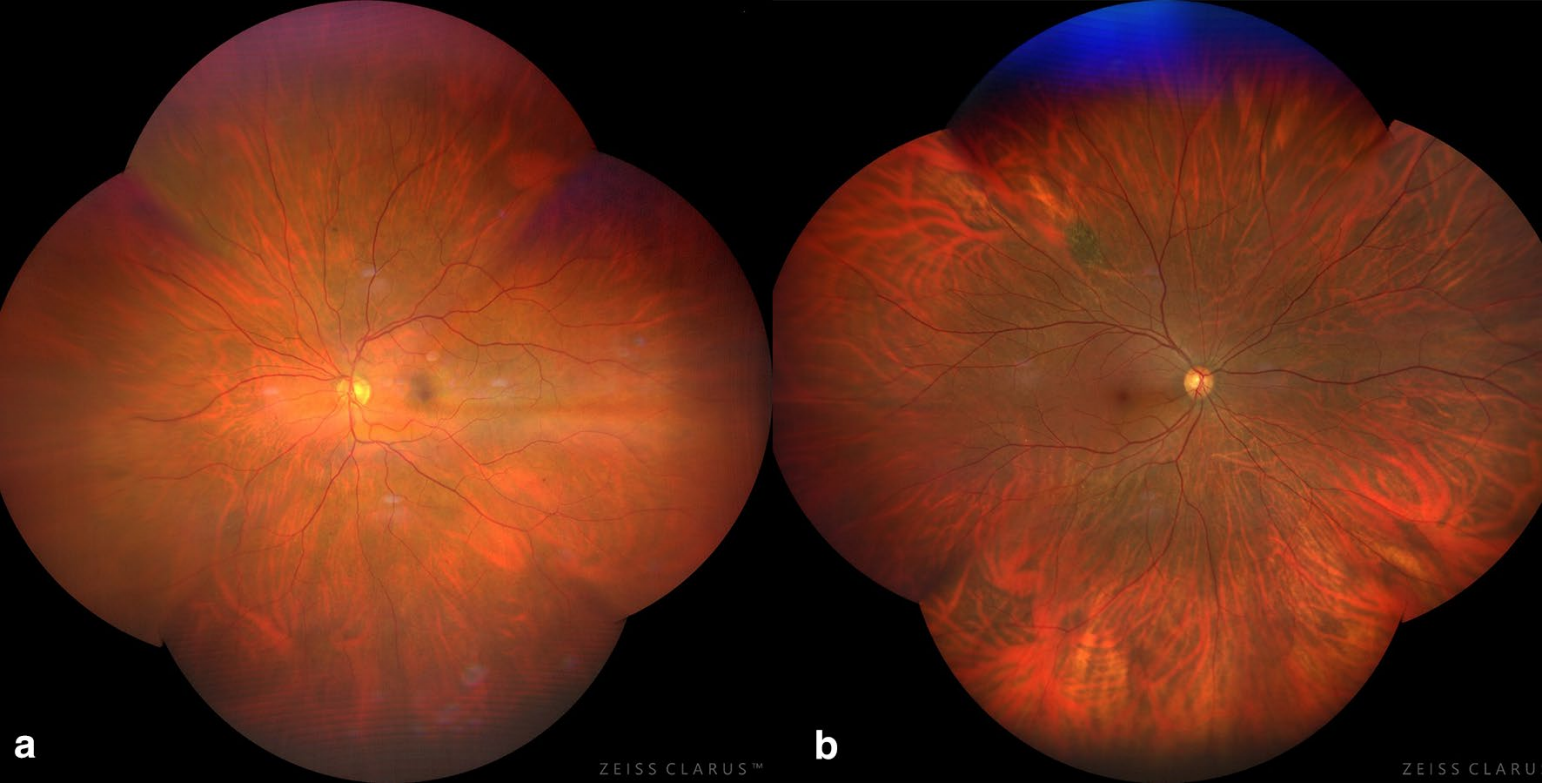
Examples of missed diagnosis with clinical examination: (**a**) Mild diabetic retinopathy; (**b**) small, flat nevus.

A total of 7250 eyes of 3625 patients were consecutively enrolled. 226 eyes were excluded due to significant media opacities (172) and/or poor quality images (54). 7024 eyes were finally examined. The clinical features (CG) of the eyes examined were the following: diabetic retinopathy, from mild to proliferative, occurred in 1819 eyes (26% of the total) (Fig. 2A); vascular abnormalities, such as vascular occlusions, Coats disease or hemangiomas, affected 380 eyes (5%); macular degenerations (age-related, central serous chorioretinopathy) or hereditary dystrophies appeared in 777 eyes (11%); retinal or choroidal tumors such choroidal melanoma, retinoblastoma, choroidal metastases were found in 1426 eyes (20%) (Fig. 2B); peripheral lesions, namely: retinal detachment, retinal breaks, benign retinal degenerations, clinically evident posterior vitreous detachment, occurred in 369 eyes (5%); myopic eyes with typical chorioretinal lesions were 550 (8%) and normal eyes were 1703 (24%). The results of clinical and imaging classifications are reported in Table 1. The time required for each clinical evaluation was of 8.5 ± 3.0 min, 3.0 ± 1.5 for each auto-montage imaging acquisition, 3.5 ± 1.0 for imaging analysis.

**Table 1:** Clinical grading versus Imaging grading.

Retinal disorders	Clinical Grading	Imaging grading 1	Imaging grading 2
Diabetic Retinopathy	1819	1816	1820
Vascular Abnormalities	380	383	379
Peripheral lesions and retinal detachment	369	370	372
Choroidal and retinal tumours	1426	1429	1428
Macular degenerations and dystrophies	777	769	772
Myopic alterations	550	548	547
Normal retina	1703	1709	1706
Total	7024	7024	7024

The inter-grader agreement between clinical imaging graders was substantial (k = 0.998, 95%IC = 0.997–0.999). The analysis among clinical (CG) and imaging diagnosis (IG1, IG2) of retinal disorders showed an excellent concordance of the two methods, both in the overall results, with k = 0.997 (95%IC = 0.996–0.999), and in each individual disorder (k ≥ 0.994). PABAK and Gwet’s first-order agreement coefficient did not show any significant difference with k coefficient. Compared classifications and k index (standard and adjusted) with the 95% confidence interval assessed for each disorder are reported in Table 2.

**Table 2:** Comparison of classifications.

Classifications	+ +	+ −	− +	−	p_0_	BI	PI	k (95%IC)	PABAK(95%IC)
**IMG2 vs IMG1**									
Diabetic retinopathy	1816	4	0	5204	0.9994	0.0006	0.4823	0.9985 (0.9971–1.0000)	0.9989 (0.9977–1.0000)
Vascular abnormalities	379	0	4	6641	0.9994	0.0006	0.8915	0.9944 (0.9890–0.9999)	0.9989 (0.9977–1.0000)
Peripheral alterations and retinal detachment	370	2	0	6652	0.9997	0.0003	0.8944	0.9972 (0.9932–1.0000)	0.9994 (0.9986–1.0000)
Tumors	1428	0	1	5595	0.9999	0.0001	0.5933	0.9996 (0.9987–1.0000)	0.9997 (0.9992–1.0000)
Macular degenerations and dystrophies	769	3	0	6252	0.9996	0.0004	0.7806	0.9978 (0.9953–1.0000)	0.9991 (0.9982–1.0000)
Myopic alterations	547	0	1	6476	0.9999	0.0001	0.8441	0.9990 (0.9971–1.0000)	0.9987 (0.9992–1.0000)
Normal	1706	0	3	5315	0.9996	0.0004	0.5138	0.9988 (0.9975–1.0000)	0.9991 (0.9982–1.0000)
**CG vs IMG1**									
Diabetic retinopathy	1816	3	0	5205	0.9996	0.0004	0.4825	0.9989 (0.9976–1.0000)	0.9991 (0.9982–1.0000)
Vascular abnormalities	380	0	3	6641	0.9996	0.0004	0.8914	0.9958 (0.9911–1.0000)	0.9991 (0.9982–1.0000)
Peripheral alterations and retinal detachment	369	0	1	6654	0.9999	0.0001	0.8948	0.9986 (0.9958–1.0000)	0.9997 (0.9992–1.0000)
Tumors	1426	0	3	5595	0.9996	0.0004	0.5935	0.9987 (0.9972–1.0000)	0.9991 (0.9982–1.0000)
Macular degenerations and dystrophies	769	8	0	6247	0.9989	0.0011	0.7799	0.9942 (0.9902–0.9982)	0.9977 (0.9961–0.9993)
Myopic alterations	548	2	0	6474	0.9997	0.0003	0.8437	0.9980 (0.9953–1.0000)	0.9994 (0.9986–1.0000)
Normal	1703	0	6	5315	0.9991	0.0009	0.5142	0.9977 (0.9958–0.9995)	0.9983 (0.9969–0.9997)
**CG vs IMG2**									
Diabetic retinopathy	1819	1	0	5204	0.9999	0.0001	0.4819	0.9996 (0.9989–1.0000)	0.9997 (0.9992–1.0000)
Vascular abnormalities	379	1	0	6644	0.9999	0.0001	0.8919	0.9986 (0.9959–1.0000)	0.9997 (0.9992–1.0000)
Peripheral alterations and retinal detachment	369	0	3	6652	0.9996	0.0004	0.8945	0.9957 (0.9909–1.0000)	0.9991 (0.9982–1.0000
Tumors	1426	0	2	5596	0.9997	0.0003	0.5937	0.9991 (0.9979–1.0000)	0.9994 (0.9986–1.0000)
Macular degenerations and dystrophies	772	5	0	6247	0.9993	0.0007	0.7795	0.9964 (0.9932–0.9996)	0.9986 (0.9973–0.9998)
Myopic alterations	547	3	0	6474	0.9996	0.0004	0.8438	0.9970 (0.9937–1.0000)	0.9991 (0.9982–1.0000)
Normal	1703	0	3	5318	0.9996	0.0004	0.5147	0.9988 (0.9975–1.0000)	0.9991 (0.9982–1.0000)

*CG Clinical grader classification; *IMG1* Image grader 1 classification; *IMG2* Image grader classification; +  +  = eyes classified with a specific disorder correctly by both examiners; +  − / −  +  = eyes classified correctly by one examiner; −  = eyes classified without the specific disorder by both examiner; p_0_ = observed agreement; *BI* bias index; *PI* prevalence index; k (95%IC) = kappa coefficient with the corresponding 95% confidence interval calculated for each disease; PABAK (95%IC) = Prevalence-adjusted and bias-adjusted kappa with the corresponding 95% confidence interval calculated for each disease.

No significant difference appeared in retinal evaluation between the two procedures. An overall of 5316 of 5321 eyes were correctly classified by UWF imaging method, showing a sensibility and specificity of almost 100% for each disease group. See Table 3 for sensibility and specificity results in individual groups.

**Table 3:** Sensibility and specificity in individual diseases.

Classifications	+ +	+ −	− +	–	SS	SP
**CG vs IMG1**						
Diabetic retinopathy	1816	3	0	5205	100.00	99.94
Vascular abnormalities	380	0	3	6641	99.22	100.00
Peripheral alterations and retinal detachment	369	0	1	6654	99.73	100.00
Tumors	1426	0	3	5595	99.79	100.00
Macular degenerations and dystrophies	769	8	0	6247	100.00	99.87
Myopic alterations	548	2	0	6474	100.00	99.97
Normal	1703	0	6	5315	99.65	100.00
**CG vs IMG2**						
Diabetic retinopathy	1819	1	0	5204	100.00	99.98
Vascular abnormalities	379	1	0	6644	100.00	99.98
Peripheral alterations and retinal detachment	369	0	3	6652	99.19	100.00
Tumors	1426	0	2	5596	99.86	100.00
Macular degenerations and dystrophies	772	5	0	6247	100.00	99.92
Myopic alterations	547	3	0	6474	100.00	99.95
Normal	1703	0	3	5318	99.82	100.00

*CG Clinical grader classification; *IMG1* Image grader 1 classification; *IMG2* Image grader classification; +  +  = eyes classified with a specific disorder correctly by both examiners; +  − / −  +  = eyes classified correctly by one examiner;− = eyes classified without the specific disorder by both examiner; *SS* sensibility; *SP* specificity.

## Discussion

Although indirect ophthalmoscopy, particularly associated to scleral depression, and posterior biomicroscopy still remain the gold standard for fundus examination^19^, over the last ten years the use of fundus photography system has extensively increased^11,12,20^, as an adjunct to clinical evaluation^7,21,22^ or as a screening tool for many retinal diseases, in particular diabetic retinopathy^4–6,10^, peripheral retinal lesions^8,9^ and other retinal disorders^16,23,24^, also by means of deep learning technologies^3,6,25,26^. This is due to the progressive improvement of retinal fundus cameras, involving both the extent of retinal field evaluated and the quality of images: from the earliest cameras encompassing a retinal field of 20–30° in a single image ^12^, we have now systems capable of acquiring real color fundus images covering up to 200° of retinal field with a pixel definition of 6604 × 4274 (Clarus™), or providing a two-tone fundus image of 200° of field with a 3900 × 3072 pixel definition (Optos®)^4,12,14^. The COVID-19 pandemic has exacerbated this trend due to the need for reducing close contacts and the burden on health-care systems^1–3^: besides safety, eye tele-screening reduces the time of the examination both in adults and in children^3^.

Recent studies evaluated UWF imaging for individual disorders, most of which performed on diabetic retinopathy, peripheral retinal lesions or other retinal disorders. Some reports have shown that UWF fundus imaging is an effective useful tool for the assessment of diabetic retinopathy^4,5,12^, focusing on the higher accuracy of Clarus™ in detecting microaneurysm and retinal hemorrhages: by providing real color images and reducing lids and lashes artifacts, it allows a slightly more precise staging of diabetic retinopathy and maculopathy than Optos® camera^4^. Conversely, studies assessing the effectiveness of UWF imaging in detecting peripheral retinal lesions, such as retinal degenerations, retinal breaks, rhegmatogenous retinal detachment, have been conducted primarily with Optos®: despite considering UWF imaging a useful adjunct to medical evaluation, some authors do not agree it may represent substitute of clinical ophthalmoscopy due to the possible missing of some peripheral retinal lesions^7,21^. Other authors observed consistent findings between clinical and UWF imaging examination, and consider the two methods interchangeable^8,19^. Moreover, even if the effective fields of views between Clarus™ and Optos® seem different depending on the specific retinal quadrant^14^, a recent study found a similar ability to detect treatment-requiring retinal breaks between the two systems^9^. UWF imaging appeared to be useful also for screening of ocular Toxoplasmosis^23^ and even superior to dilated fundus examination for the screening of sickle cell retinopathy, because of the higher accuracy in detecting capillary occlusion or anastomosis^16^, and in inherited retinal dystrophies, providing previously unavailable information about retinal periphery^24^.

Our study was planned to compare retinal UWF imaging versus clinical evaluation and to validate the technique of clinical analysis of retinal UWF imaging, obtained by means of Clarus™, performed without a deep learning system. We enrolled patients evaluated in daily clinics for presumed major retinal disorders in a period of about two years, without differentiating for individual disorder, thus collecting a very large unselected population (7024 eyes).

Our results demonstrated that UWF imaging, by means of Clarus™ fundus color images, is comparable to ophthalmoscopic examination performed with indirect ophthalmoscopy plus macular biomicroscopy, when the macula appears to be involved. For major retinal diseases these evaluation systems have similar sensitivity and specificity (almost 100%) and no statistically significant difference was found in fundus assessment between the two procedures (k = 0.997, 95%CI = 0.996–0.999). These findings differ from some previous reports^7,9,21^, but it must be underlined that the other study populations were small and mostly limited to peripheral retinal degenerations^7,9,21^. Moreover, these last reports used Optos® system^7,21^, whose limitations, mainly concerning true color, have already been reported^4^. On the other hand, our results are consistent with some recent studies^4,5,8,16,19,23^, even if these studies were also performed on small populations, for selected diagnosis (diabetic retinopathy^4,5^, peripheral lesions^8^, rhegmatogenous retinal detachment^19^, ocular Toxoplasmosis^23^, sickle cell retinopathy^16^). In accordance with another analysis ^14^, the missing diagnosis we reported were very limited in number and due to lesions located in the extreme upper temporal periphery and statistically no significant. This seem to be related to the specific examined quadrant by the limitation induced by the patient’s nose when trying to capture images of the extreme temporal periphery, since most of the missed lesions were located in the upper temporal periphery (4 cases)^9^. The classification and diagnosis achieved with UWF imaging allowed to address the patient to the correct management, such as adequate follow-up or, when needed, directing the patient to a specific clinical care pathway for the treatment of its specific retinal disorder.

The relevance of this clinical study also lies in analyzing UWF fundus photographs of such a broad population, both in terms of numbers and disorders. Moreover, the use of Clarus™ system, providing real color fundus imaging, allows realistic and accurate evaluation, comparable to the clinical one with ophthalmoscopy, except for some rare cases. However, according to patients’ symptoms and clinical features and/or to UWF photos characteristics, the examination also with indirect ophthalmoscopy may be necessary for an accurate diagnosis. In fact, at present, the approach to patients affected by retinal diseases should always be multimodal, and UWF fundus photo might be the first diagnostic modality in this approach, followed by the other, eventually clinically-indicated, diagnostic procedures, including ophthalmoscopy. Therefore, the perspective might be a shift from a “photo-assisted ophthalmoscopy” to an “ophthalmoscopy-assisted UWF fundus photography” approach.

A possible limitation of our study may be the use of this system without a deep learning tool, which may offer a more standardized classification.

In conclusion, we reported a substantial agreement in the classification of major retinal diseases using UWF imaging. This assessment highlights the interchangeability of UWF imaging and ophthalmoscopy by validating the technique of UWF imaging analysis in diagnosing major retinal diseases in daily clinical practice and should encourage the use of UWF imaging for fundus examination both in clinical and telehealth contexts. UWF imaging may improve the quality of clinical evaluation, allowing to compare images of the same eye acquired in different moments thus helping to monitor chronic diseases, such as diabetic retinopathy, and easily detect recurrences (i.e. choroidal and retinal tumors), also by using some tools intrinsic to the system. Moreover, it allows an accurate assessment of specific retinal areas, such as the macula or very peripheral sectors, without increasing the discomfort of the patient, as may happen during a prolonged ophthalmoscopic evaluation. The validation of UWF imaging for retinal evaluation also widens the possible uses of teleophthalmology: it allows to examine images at a different location than the clinic, possibly applying a deep learning algorithm, to reduce the time of evaluation and to perform screening for major retinal diseases thus rapidly directing the patient to a specific clinical care pathway if needed.

## Data Availability

The data presented in this study are available in the article. Eventual additional data are available on request from the corresponding author.
